# Deep brain stimulation of the medial forebrain bundle - effects on suicidality in treatment-refractory MDD patients?

**DOI:** 10.1192/j.eurpsy.2023.145

**Published:** 2023-07-19

**Authors:** J. C. Soares

**Affiliations:** Psychiatry and Behavioral Sciences, University of Texas Health Sciences Center at Houston, Houston, United States

## Abstract

**Abstract:**

Deep brain stimulation (DBS) of brain circuits involved in mood regulation is a novel tool that is being investigated as a potential treatment for some of the most severe patients with mood disorders. At UTHealth Houston, we have had an ongoing clinical trial with DBS of the medial forebrain bundle (MFB).

Our preliminary results suggest effects in alleviating depressive symptoms in this very severe, refractory patient group.

In this presentation, we will review our latest findings, and will also discuss the potential effects of the MFB stimulation on measures of suicidality in this patient population.

**Disclosure of Interest:**

None Declared

